# Prognostic nutritional index and mortality in pneumonia: a retrospective cohort study in China

**DOI:** 10.3389/fnut.2025.1660457

**Published:** 2025-10-15

**Authors:** Zhengrong Ding, Yunxue He, Xue Guo, Ruirui Feng, Guangqin Ren, Lili Deng, Chunjiao Zhou, Huali Tang, Zhiwei Li, Cong Zhou, Bin Li, Longdan Li

**Affiliations:** ^1^The Tenth Clinical Medical College, Guangzhou University of Traditional Chinese Medicine, Zhongshan, China; ^2^Zhongshan Hospital of Traditional Chinese Medicine Affiliated to Guangzhou University of Traditional Chinese Medicine, Zhongshan, China; ^3^Zhongshan Hospital of Traditional Chinese Medicine, Zhongshan, China; ^4^Guangzhou University of Chinese Medicine, Guangzhou, China; ^5^The Second Affiliated Hospital of Guangzhou University of Chinese Medicine (Guangdong Provincial Hospital of Chinese Medicine), Guangzhou, China; ^6^Information Engineering University of the People’s Liberation Army Cyberspace Force, Zhengzhou, China

**Keywords:** pneumonia, prognostic nutritional index, all-cause mortality, inflammatory biomarkers, nutritional-immune status

## Abstract

**Objective:**

This study aimed to investigate the association between prognostic nutritional index (PNI) and mortality risk in Chinese patients with pneumonia.

**Methods:**

This retrospective cohort study was conducted using a multicenter hospital database of adult patients with pneumonia in China. We analyzed data from 635 patients diagnosed with pneumonia at six secondary and tertiary academic hospitals in China between 1 January 2013 and 31 December 2019. Cox regression analysis was used to compare the mortality rates across the PNI tertiles. Restricted cubic spline (RCS) models, Kaplan–Meier survival analysis and Subgroup Analysis were used to explore the association between PNI and the clinical outcomes of these pneumonia patients.

**Results:**

A total of 635 patients were included. In the fully adjusted model, each 1-unit increase in PNI was associated with a 5.0% reduction in 30-day mortality risk (HR = 0.950, 95% CI: 0.929–0.972, *p* < 0.001) and a 4.5% reduction in 90-day mortality risk (HR = 0.955, 95% CI: 0.934–0.975, p < 0.001). Compared with the lowest PNI tertile (Tertile 1), patients in the highest tertile (Tertile 3) had a 64.5% lower risk of 30-day mortality (HR = 0.335, 95% CI: 0.212–0.594, *p* < 0.0001) and a 60.6% lower risk of 90-day mortality (HR = 0.394, 95% CI: 0.247–0.627, p < 0.0001). Restricted cubic spline (RCS) analysis further illustrated a consistent inverse relationship between PNI and mortality risk. Additionally, Kaplan–Meier survival curves indicated significantly lower cumulative mortality with higher PNI values.

**Conclusion:**

Our investigation identified a significant association between poorer PNI scores and higher mortality in Chinese patients with pneumonia.

## 1 Introduction

Pneumonia, including COVID-19, is a significant component of lower respiratory tract infections and is the primary cause of global disease burden. It results in over 200 million infections and more than 2.1 million deaths annually, with a particularly higher risk among the elderly and individuals with compromised immune systems (1–3). In China, the disease contributes significantly to public health costs, with over 9.5 million episodes reported in 2016 and an incidence rate of 7.13 per 1,000 person-years (4). Pneumonia results from microbial invasion of the lower respiratory tract, often leading to systemic inflammation, respiratory failure, sepsis, or death (5). Despite advances in treatment and prevention (6), pneumonia, such as Hospital-acquired pneumonia (HAP) (7), ventilator-associated pneumonia (VAP) (8), healthcare associated pneumonia (HCAP) (9) and so on, remains important causes of morbidity and mortality, especially in resource-limited settings.

Malnutrition is closely linked to poor immune function and adverse clinical outcomes (10, 11). It is commonly indicated by hypoalbuminemia and lymphopenia (12, 13), both of which impair immune responses and heighten susceptibility to severe infections. Conversely, immune dysfunction can also lead to malnutrition.

The Prognostic Nutritional Index (PNI)—calculated as 10 × serum albumin (g/dL) + 0.005 × lymphocyte count (cells/mm^3^)—integrates markers of nutritional and immune status. A higher PNI value reflects better nutritional and immune status, whereas a lower PNI indicates malnutrition and impaired immune function. Initially used to assess surgical risk (14), PNI has since been used to evaluate and predictive severity and prognosis in several types of cancer (15–17), sepsis (18), and COVID-19 (19). However, its utility in predicting mortality in pneumonia remains insufficiently explored, particularly in Chinese populations, where evidence is scarce and cutoff values vary (20, 21). To date, no large-scale prospective cohort has validated the prognostic utility of the prognostic nutritional index (PNI) for 30- or 90-day mortality among Chinese adult inpatients with pneumonia. Moreover, the optimal PNI threshold for risk stratification in this population remains undefined, and its incremental predictive benefit over established severity scores (e.g., PSI and CURB-65) has yet to be quantified.

This study aimed to investigate the association between PNI and short-term mortality in hospitalized Chinese patients with pneumonia. We hypothesize that Chinese pneumonia patients with lower PNI have higher mortality rates and poorer prognosis. By identifying whether PNI can serve as a prognostic indicator in this population, the findings may support early risk stratification and guide nutritional or clinical interventions.

## 2 Methods

### 2.1 Data source

This study was based on a publicly available dataset retrieved from the Dryad Digital Repository,^1^ which originated from a multicenter retrospective cohort investigation by Li et al. (22). The original study involved hospitalized adult patients diagnosed with pneumonia who received glucocorticoid therapy, with or without additional immunosuppressive agents. The dataset comprises detailed clinical, laboratory, microbiological, treatment, and outcome data.

### 2.2 Study design and population

**Figure 1:**
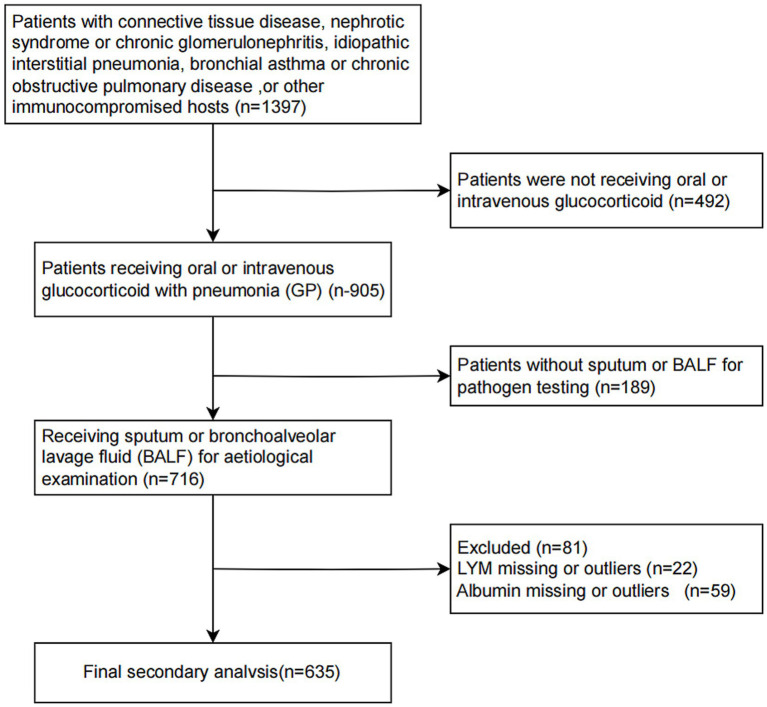
Flowchart of patient screening, eligibility, and inclusion.

This retrospective cohort study analyzed patients with available baseline nutritional and immunological parameters to calculate the PNI, and it provided information on 716 inpatients come from Six secondary and tertiary academic hospitals who were hospitalized with pneumonia between 1 January 2013 and 31 December 2019 in China (22). Patients were eligible if they met the following criteria: age ≥16 years, hospitalized with a clinical diagnosis of pneumonia, oral or intravenous glucocorticoid treatment before admission, and had available serum albumin and lymphocyte count upon admission,as detailed in Li et al. (22). Patients were excluded if they had missing data necessary to compute PNI, incomplete outcome data, or evident data irregularities (Figure 1). The primary outcomes were 30-day and 90-day all-cause in-hospital mortality. This was a secondary analysis of a multicenter dataset; no stratification or modeling by hospital site was conducted.

### 2.3 Study quality control

Data consistency, plausibility and completeness were assessed before analysis. Outliers and biologically implausible values were carefully reviewed. Patients with key missing laboratory values were excluded to avoid imputation bias. Variable definitions and units were cross-validated against the original study documentation. To reduce potential misclassification, all diagnoses, including pneumonia and comorbid conditions, were recorded based on discharge summaries validated by clinicians at participating hospitals. Mortality outcomes were confirmed through hospital records.

### 2.4 Diagnostic procedures

The guidelines from the American Thoracic Society (ATS) and the Infectious Diseases Society of America (IDSA) are followed for the diagnosis of pneumonia. The definition of pneumonia is based on clinical symptoms (such as cough, fever, and dyspnea), radiological findings consistent with pulmonary infiltrates, and the exclusion of other diagnoses. Laboratory evaluations upon admission included a complete blood count, serum albumin, C-reactive protein, creatinine levels, and microbiological tests where applicable. PNI was calculated as a continuous variable and also categorized into tertiles for subgroup comparisons based on prior literature (6, 23–26) and the actual distribution of PNI in our study population. All laboratory results were those obtained within the first 24 h of hospital admission.

### 2.5 Statistical analysis

Baseline characteristics were summarized by PNI tertiles using appropriate descriptive statistics: means ± standard deviations for normally distributed continuous variables, medians with interquartile ranges (IQR) for non-normally distributed data, and counts (percentages) for categorical variables. Group comparisons were performed using ANOVA or Kruskal–Wallis tests for continuous variables, and Chi-square tests for categorical variables. Variables exhibiting more than 10% missingness were excluded. For variables with missingness below this threshold, multivariate chained equations were used for imputation, assuming data were missing at random (MAR). Kaplan–Meier survival curves were constructed to compare cumulative survival rates across PNI tertiles, with statistical significance evaluated using the log-rank test. Cox proportional hazards regression models were employed to evaluate the association between PNI (as both continuous and categorical variables) and 30-day and 90-day mortality. Multivariable models were sequentially adjusted for demographic characteristics, comorbidities, disease severity indicators, and treatment variables. Hazard ratios (HRs) with 95% confidence intervals (CIs) were reported. Restricted cubic spline analysis was conducted to assess potential non-linear associations between PNI and mortality risk. Subgroup analyses and interaction tests were performed to evaluate the consistency of associations across clinically relevant strata. A two-sided *p*-value <0.05 was considered statistically significant. Statistical analyses were carried out using R software (http://www.R.project.org, The R Foundation) and Free Statistics software version 2.1.1.

Covariates for multivariable models were selected based on statistical significance in univariable analyses (*p* < 0.05), a change of >10% in effect estimates, and evidence from previous studies (27) and clinical relevance. To assess multicollinearity among the covariates, we employed the variance inflation factor (VIF). Covariates with a VIF exceeding 10 were considered to exhibit a high degree of collinearity and were consequently excluded from the regression model.

### 2.6 Patient and public involvement

As this study involved a secondary analysis of de-identified, publicly available data, no patients or members of the public were involved in the study design, data collection, analysis, or interpretation.

## 3 Results

### 3.1 Patient selection and characteristics

A total of 635 hospitalized patients diagnosed with pneumonia and treated with glucocorticoids were included in the final analysis. The median age was 58.3 years and 52.3% were male. Based on tertiles of the PNI, patients were categorized into Tertile 1 (PNI < 34.35), Tertile 2 (PNI:34.35 ~ 40.93), and Tertile 3 (PNI > 40.93). Baseline characteristics stratified by PNI tertiles are shown in Table 1. The overall mean PNI was 38.2 ± 8.4, with substantial variation across tertile groups.

**Table 1:** Baseline characteristics of the study participants, overall and stratified by PNI score tertiles.

Variables	Total	PNI, T1(<34.35)	PNI, T2(34.35 ~ 40.93)	PNI, T3(>40.93)	p
	(*n* = 635)	(*n* = 211)	(*n* = 212)	(*n* = 212)	
Demographic data					
Age (years), Mean ± SD	58.3 ± 16.4	58.3 ± 17.0	58.5 ± 15.5	58.1 ± 16.9	0.964
Male, *n* (%)	332 (52.3)	114 (54)	111 (52.4)	107 (50.5)	0.765
Comorbidities					
HBP, *n* (%)	221 (34.8)	77 (36.5)	75 (35.4)	69 (32.5)	0.680
DM, n (%)	164 (25.8)	51 (24.2)	42 (19.8)	71 (33.5)	0.005
Respiratory Failure, n (%)	323 (50.9)	152 (72)	98 (46.2)	73 (34.4)	< 0.001
Clinical parameters					
Temperature, Mean ± SD	37.3 ± 1.0	37.6 ± 1.1	37.2 ± 0.9	37.1 ± 0.9	< 0.001
Heart rate, Mean ± SD	88.8 ± 24.5	93.4 ± 25.4	87.0 ± 23.9	86.0 ± 23.8	0.003
Respiratory rate, Mean ± SD	23.0 ± 6.2	24.5 ± 7.9	22.7 ± 5.5	21.7 ± 4.3	< 0.001
SBP, Mean ± SD	122.6 ± 19.2	122.7 ± 21.0	123.0 ± 18.4	122.1 ± 18.2	0.884
DBP, Mean ± SD	74.5 ± 12.5	72.9 ± 12.8	75.3 ± 12.6	75.4 ± 11.9	0.058
MV, *n* (%)	226 (35.6)	113 (53.6)	61 (28.8)	52 (24.5)	< 0.001
Invasive ventilation, *n* (%)	153 (24.1)	71 (33.6)	46 (21.7)	36 (17.0)	< 0.001
Noninvasive ventilation, *n* (%)	150 (23.6)	77 (36.5)	39 (18.4)	34 (16.0)	< 0.001
PSI, Mean ± SD	81.0 ± 31.2	91.0 ± 31.7	79.1 ± 30.5	72.9 ± 28.7	< 0.001
PNI, Mean + SD	38.2 ± 8.4	29.6 ± 3.7	37.7 ± 1.8	47.2 ± 6.5	< 0.001
Laboratory parameters					
LAC, Median (IQR)	1.0 (0.0, 1.0)	1.0 (0.0, 1.0)	1.0 (0.0, 1.0)	0.0 (0.0, 1.0)	< 0.001
WBC (×10^9^/L), Mean ± SD	9.2 ± 5.5	8.8 ± 4.7	8.6 ± 4.1	10.1 ± 7.1	0.011
NUET (×10^9^/L), Median (IQR)	6.6 (4.3, 10.1)	7.1 (4.7, 10.9)	6.4 (4.3, 9.2)	6.2 (3.9, 10.0)	0.075
LYM (×10^9^/L), Median (IQR)	0.8 (0.5, 1.4)	0.5 (0.3, 0.8)	0.8 (0.6, 1.2)	1.4 (1.0, 2.1)	< 0.001
HGB (g/L), Mean ± SD	112.7 ± 22.1	104.6 ± 20.4	113.6 ± 21.2	119.9 ± 22.2	< 0.001
PLT (×10^9^/L), Mean ± SD	191.1 ± 90.0	173.5 ± 98.0	193.3 ± 87.8	206.4 ± 80.9	< 0.001
ALB (g/L), Mean ± SD	32.8 ± 6.4	26.7 ± 3.7	33.1 ± 2.7	38.4 ± 5.7	< 0.001
AST(μ/L), Median (IQR)	24.0 (16.0, 39.1)	29.0 (17.4, 52.5)	24.0 (16.6, 38.0)	20.2 (15.0, 30.0)	< 0.001
ALT(μ/L), Median (IQR)	24.0 (15.0, 45.0)	25.0 (15.5, 49.0)	24.0 (16.0, 45.0)	21.6 (14.0, 40.2)	0.208
BUN (mg/dL), Median (IQR)	6.3 (4.7, 9.8)	7.4 (5.4, 11.9)	6.0 (4.5, 9.0)	5.9 (4.4, 9.0)	< 0.001
Cr(mg/dL), Median (IQR)	64.0 (51.0, 89.4)	73.9 (50.9, 110.0)	60.8 (50.0, 81.8)	62.9 (51.6, 80.0)	0.006
K^+^ (mmol/L), Median (IQR)	3.9 (3.6, 4.2)	3.8 (3.5, 4.2)	4.0 (3.6, 4.2)	3.9 (3.6, 4.2)	0.064
Na^+^ (mmol/L), Mean ± SD	137.5 ± 6.6	135.4 ± 5.6	137.6 ± 5.0	139.5 ± 8.1	< 0.001
Treatment measures					
Vasoactive drugs, *n* (%)	110 (17.3)	57 (27.0)	31 (14.6)	22 (10.4)	< 0.001
High-dose glucocorticoid, *n* (%)	228 (35.9)	100 (47.4)	69 (32.5)	59 (27.8)	< 0.001
Immunosuppressant, *n* (%)	262 (41.3)	90 (42.7)	86 (40.6)	86 (40.6)	0.881
Ganciclovir, *n* (%)	306 (48.2)	118 (55.9)	97 (45.8)	91 (42.9)	0.019
Sulfonamide, *n* (%)	303 (47.7)	114 (54.0)	106 (50.0)	83 (39.2)	0.007
Anti-Aspergillus, *n* (%)	254 (40.0)	106 (50.2)	83 (39.2)	65 (30.7)	< 0.001
Anti-Pseudomonas, *n* (%)	486 (76.5)	184 (87.2)	161 (75.9)	141 (66.5)	< 0.001
Outcome					
Status 30-day, *n* (%)	144 (22.7)	84 (39.8)	38 (17.9)	22 (10.4)	< 0.001
Status 90-day, *n* (%)	166 (26.1)	92 (43.6)	46 (21.7)	28 (13.2)	< 0.001

PNI, Prognostic Nutritional Index, HBP, Hypertension; DM, Diabetes mellitus; SBP, Systolic pressure; DBP, Diastolic pressure; MV, Mechanical Ventilation; PSI, Pneumonia Severity Index; LAC, Lactate; WBC, White blood cell; NUET, Neutrophils; LYM, Lymphocyte; HGB, Hemoglobin; PLT, Platelet; ALB, Albumin; AST, Aspartate aminotransferase; ALT, Alanine aminotransferase; BUN, Blood Urea Nitrogen; Cr, Creatinine; K^+^, Potassium; Na^+^, Sodium.

Compared with patients in Tertile 3, those in Tertile 1 had significantly higher respiratory and heart rates, lower serum albumin and lymphocyte counts, and a higher incidence of invasive mechanical ventilation and septic shock (all *p* < 0.05). The 30-day and 90-day mortality rates were also significantly higher in Tertile 1 group.

### 3.2 Association between PNI and short-term mortality

In univariate Cox regression analysis Supplementary Table S1, lower PNI was associated with increased risk of both 30-day and 90-day mortality.

Multivariable Cox regression analysis results are presented in Table 2. When modeled as a continuous variable, the PNI showed a significant inverse association with both 30-day and 90-day mortality across all models. In the fully adjusted model (Model IV), each 1-unit increase in PNI was associated with a 5.0% reduction in 30-day mortality risk (HR = 0.950, 95% CI: 0.929–0.972, *p* < 0.001) and a 4.5% reduction in 90-day mortality risk (HR = 0.955, 95% CI: 0.934–0.975, *p* < 0.001). For categorical PNI, we emphasized the contrast between the highest (Tertile 3) and lowest tertiles (Tertile 1). Patients in Tertile 3 had approximately 64.5% lower 30-day mortality risk (HR = 0.355, 95% CI: 0.212–0.594, *p* < 0.0001) and 60.6% lower 90-day mortality risk (HR = 0.394, 95% CI: 0.247–0.627, *p* < 0.0001) compared with Tertile 1.

**Table 2:** Multivariable COX regression analysis of 30-day and 90-day mortality.

Exposure	30-d all-cause death								90-d all-cause death							
	Model I		Model II		Model III		Model IV		Model I		Model II		Model III		Model IV	
	HR (95% CI)	*P*-value	HR (95% CI)	*P*-value	HR (95% CI)	*P*-value	HR (95% CI)	*P*-value	HR (95% CI)	*P*-value	HR (95% CI)	*P*-value	HR (95% CI)	*P*-value	HR (95% CI)	*P*-value
PNI	0.925 (0.905 ~ 0.946)	<0.001	0.925 (0.905 ~ 0.946)	<0.001	0.952 (0.932 ~ 0.973)	<0.001	0.950 (0.929 ~ 0.972)	<0.001	0.930 (0.911 ~ 0.950)	<0.001	0.930 (0.911 ~ 0.950)	<0.001	0.958 (0.939 ~ 0.977)	<0.001	0.955 (0.934 ~ 0.975)	<0.001
PNI Tertile 1	1(Ref)		1(Ref)		1(Ref)		1(Ref)		1(Ref)		1(Ref)		1(Ref)		1(Ref)	
PNI Tertile 2	0.385 (0.263 ~ 0.566)	<0.001	0.384 (0.262 ~ 0.563)	<0.001	0.569 (0.384 ~ 0.842)	0.0049	0.625 (0.417 ~ 0.937)	0.023	0.418 (0.293 ~ 0.595)	<0.001	0.416 (0.292 ~ 0.593)	<0.001	0.626 (0.435 ~ 0.9)	0.0114	0.691 (0.475 ~ 1.006)	0.054
PNI Tertile 3	0.213 (0.133 ~ 0.34)	<0.001	0.211 (0.132 ~ 0.338)	<0.001	0.348 (0.209 ~ 0.58)	< 0.0001	0.355 (0.212 ~ 0.594)	< 0.0001	0.241 (0.158 ~ 0.368)	<0.001	0.239 (0.157 ~ 0.365)	<0.001	0.403 (0.255 ~ 0.638)	< 0.0001	0.394 (0.247 ~ 0.627)	< 0.0001
*P* for trend		<0.001		<0.001		<0.001		<0.001		<0.001		<0.001		<0.001		< 0.0001

Model I: Non-adjusted model; Model II: Adjusted for Age and Gender; Model III: Model II + Respiratory Failure, DM, LAC, WBC, HGB, ALT, Cr; Model IV: Model III + MV, PSI, Vasoactive drugs, High-dose glucocorticoid, Ganciclovir, Sulfonamide, Anti-Aspergillus, Anti-Pseudomonas. PNI, Prognostic Nutritional Index; DM, Diabetes mellitus; LAC, Lactate; WBC, White blood cell; HGB, Hemoglobin; ALT, Alanine aminotransferase; Cr, Creatinine; MV, Mechanical Ventilation; PSI, Pneumonia Severity Index.

### 3.3 Restricted cubic spline model and Kaplan–Meier survival analysis

**Figure 2:**
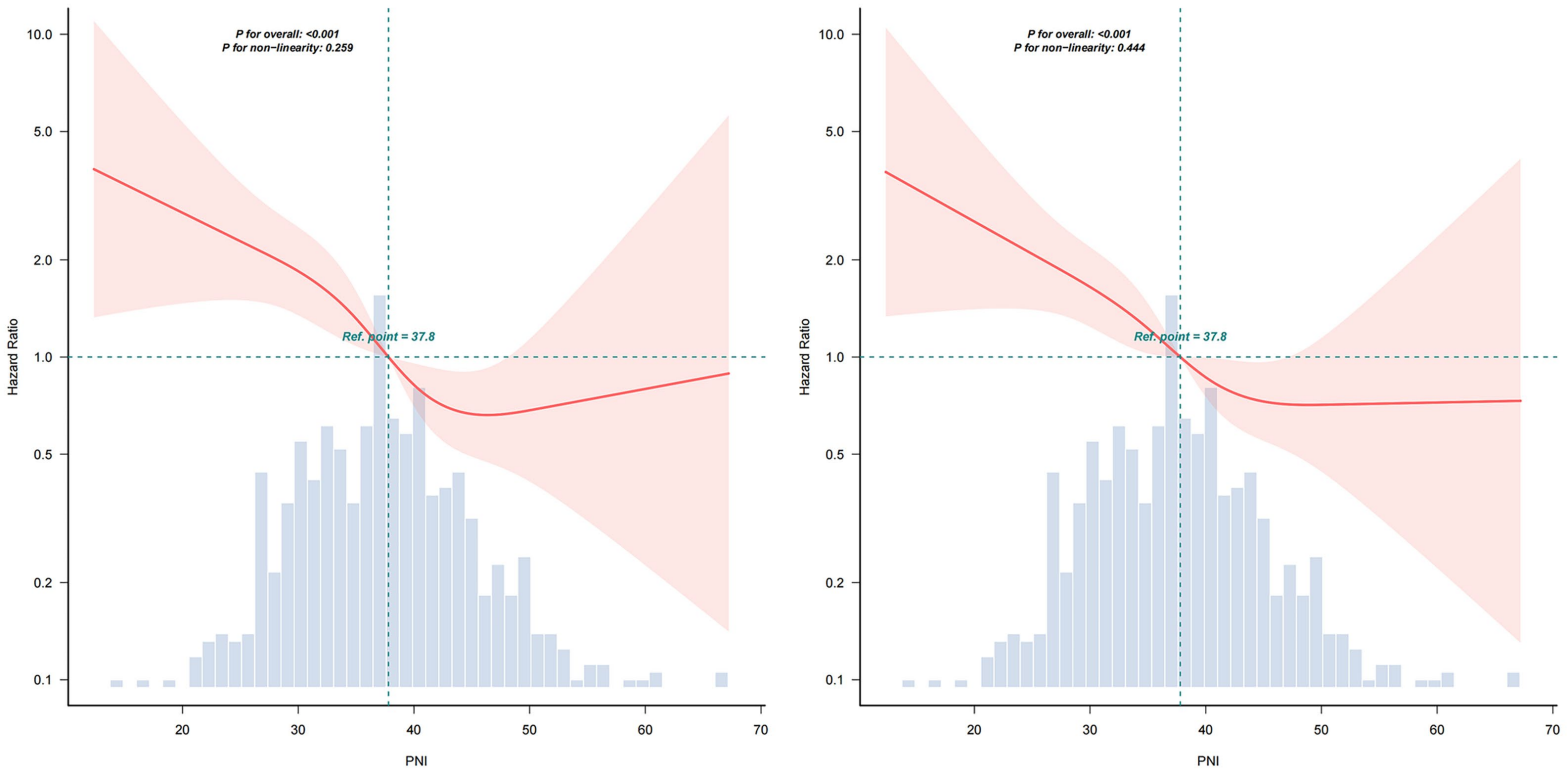
Restricted cubic spline model of the PNI for 30-day (left) and 90-day (right) mortality. Solid lines represent the estimated hazard ratios (HRs) for mortality across the continuous spectrum of PNI, and the shaded areas indicate 95% confidence intervals (CIs). PNI, Prognostic Nutritional Index.

The restricted cubic spline model in Figure 2 revealed a significant relationship between PNI and 30-day (A: p for overall <0.001, p for non-linearity = 0.089) and 90-day mortality risk (B: p for overall <0.001, p for non-linearity = 0.08) after adjusted for Age, Gender, Respiratory Failure, DM, LAC, WBC, HGB, ALT, Cr, MV, PSI, Vasoactive drugs, High-dose glucocorticoid, Ganciclovir, Sulfonamide, Anti-Aspergillus, Anti-Pseudomonas. The HR of mortality decreased sharply as PNI values rose. The shaded area around the curve represents the 95% confidence interval, which provides an estimate of the precision of the hazard ratio estimates.

**Figure 3:**
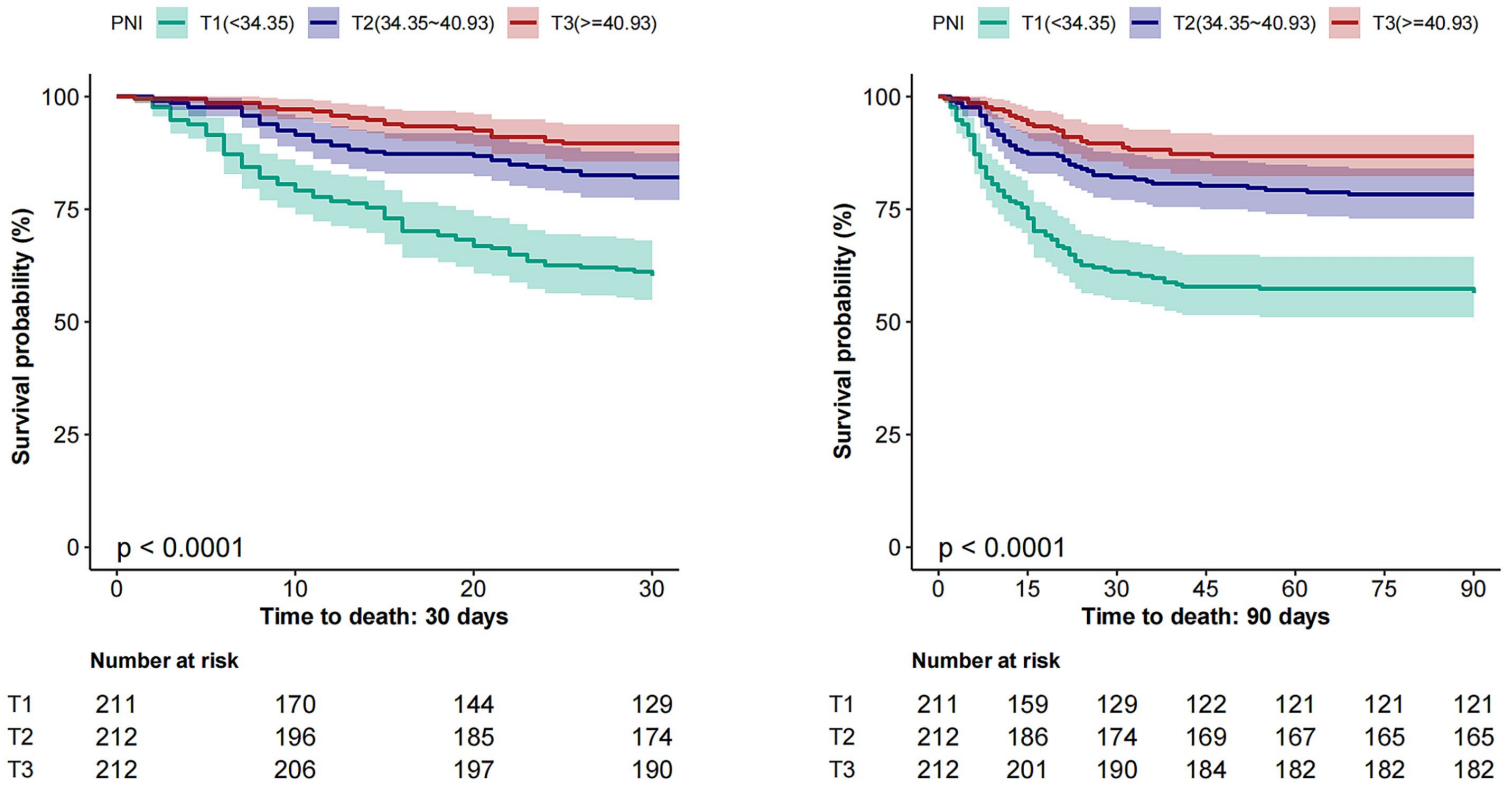
Kaplan–Meier survival curves for 30-day (Left) and 90-day (Right) mortality by PNI tertiles. PNI, Prognostic Nutritional Index.

Kaplan–Meier curves illustrate progressively lower cumulative survival probabilities in patients with lower PNI levels over both 30-day and 90-day follow-up periods. The survival differences across the tertiles were statistically significant, as determined by the log-rank test (*p* < 0.001 for both endpoints) (see Figure 3).

### 3.4 Subgroup analysis

**Figure 4:**
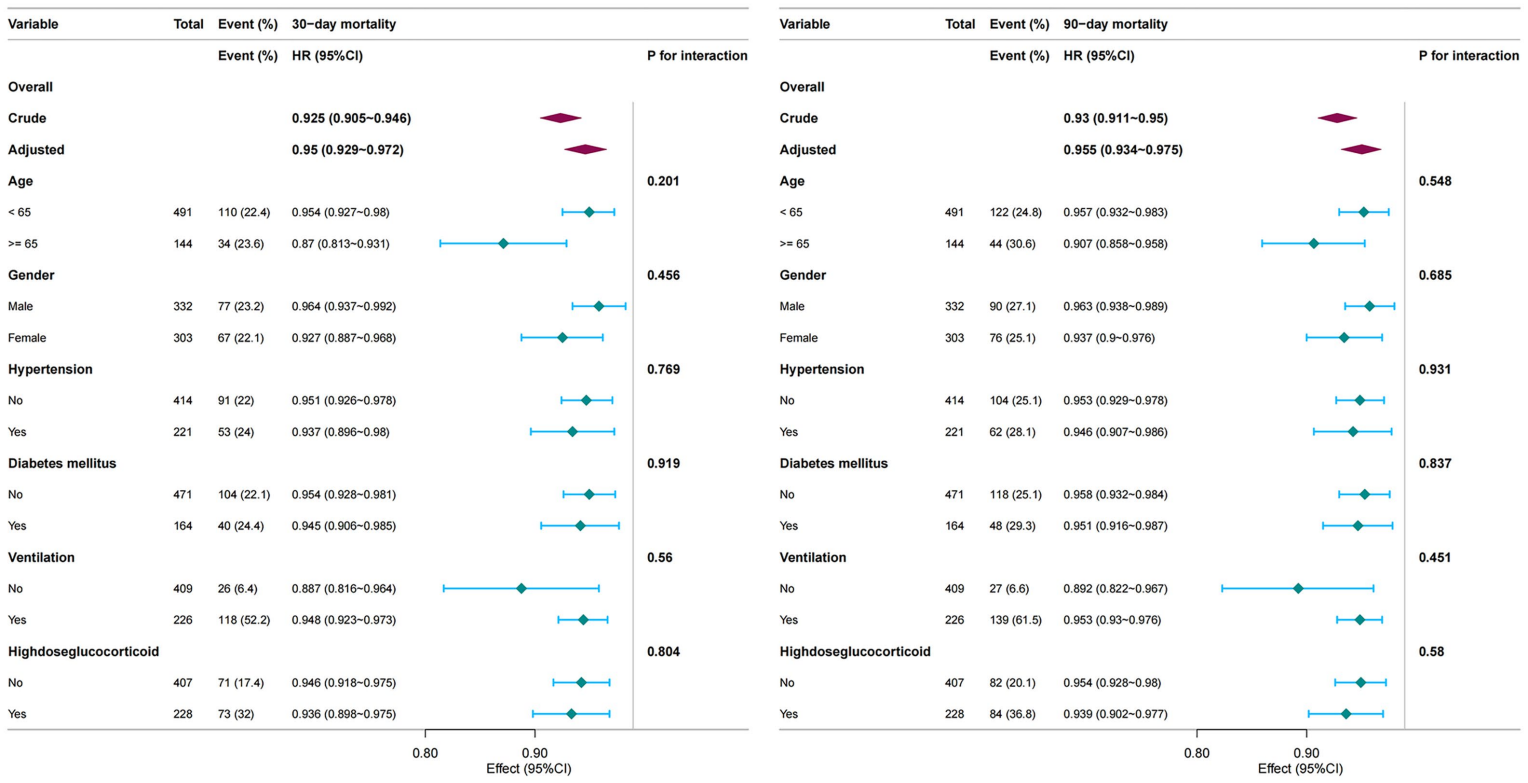
Subgroup analyses of the association between PNI and short-term mortality. PNI Prognostic Nutritional Index.

Subgroup analyses were conducted to evaluate the consistency of the association between PNI and mortality across strata of Age, Gender, Hypertension, Diabetes mellitus, Mechanical Ventilation, High-dose glucocorticoid (Figure 4). The inverse relationship between PNI and mortality remained robust across all subgroups, with no significant interactions observed (p for interaction > 0.05). These subgroup findings are descriptive, and no adjustment for multiple comparisons was performed.

## 4 Discussion

Pneumonia has imposed a significant disease burden on society, and the lack of direct and effective biomarkers for prognosis continues to pose a challenge. Additionally, there are limited studies on the relationship between the PNI and pneumonia patients in China. Our analysis, based on a cohort of 635 Chinese pneumonia patients, revealed substantial 30-day and 90-day mortality rates of 22.7 and26.1%, respectively. After adjusting for confounders, PNI showed a significant negative association with 30-day mortality [HR, 0.950 (0.929–0.972), *p* < 0.001] and 90-day mortality [HR, 0.955 (0.934–0.975), *p* < 0.001]. The PNI demonstrated predictive value for 30-day and 90-day mortality in Chinese pneumonia patients, with AUCs of 0.699 and 0.686, respectively (Supplementary Figure S1). These findings align with the pathophysiological interplay between hypoalbuminemia—a marker of catabolic stress and endothelial dysfunction—and lymphopenia, which reflects impaired adaptive immunity (28). However, given the number of subgroup analyses conducted, the possibility of false-positive results due to multiple comparisons cannot be excluded. These findings should therefore be interpreted with caution and confirmed in future prospective studies.

Albumin, as the most abundant plasmatic protein, plays a crucial role in a variety of physiological processes, these functions include maintaining plasma colloid osmotic pressure, providing antioxidation and anticoagulation benefits, regulating immune functions, and protecting the integrity of vascular walls (29, 30). One study has demonstrated that severe hypoalbuminemia is associated with the cytokine storm induced by COVID-19, which is related to the intensification of disease-related inflammatory responses and progression of the illness, ultimately culminating in the fatality of some severely ill patients (31). A research about 289 patients of acute respiratory diseases showed that low serum albumin level was associated to one, two- and five-year mortality after hospital stay (all *p* < 0.05) (32). The study of Zhao et al. demonstrated that serum albumin (ALB) is an independent prognostic variable for 30-day survival in patients with community-acquired pneumonia (CAP), and that albumin is negatively correlated with the Pneumonia Severity Index (PSI) (33). Albumin is widely used as an indicator of malnutrition (34).

In addition to albumin, another component of PNI, lymphocyte count, also plays a critical role in immunity. Similarly, lymphocyte count acts as a pivotal indicator of cellular immunity, Lymphopenia has long been implicated as a potential biomarker for acute infection (35). A decline in lymphocyte counts, primarily attributed to heightened adhesion, redistribution, and expedited apoptosis, is indicative of weakened immune responses (36). Although the exact mechanisms remain incompletely understood, lymphopenia is considered to result from enhanced apoptosis, redistribution of lymphocytes to lymphoid organs or sites of infection, and diminished lymphopoiesis (37). Lymphopenia (a reduction in the normal concentration of lymphocytes) is prevalent in many patients with pneumonia (35, 38), several studies suggested that lymphopenia has been linked to an increased risk of mortality in pneumococcal pneumonia (13), COVID-19 (39), primary care pneumonia (35), and ICU-acquired pneumonia (40). And these studies suggest that lymphopenia during or at the onset of infection is closely correlated with adverse clinical outcomes.

Considering the complexity of pneumonia’s clinical course, which is influenced by multiple factors, relying on a single predictive indicator often proves insufficient for accurately forecasting clinical outcomes. Serum albumin and lymphocyte count, the two key components of the prognostic nutritional index (PNI), capture complementary aspects of nutritional and immune status. Albumin reflects protein reserve and exerts antioxidant and anti-inflammatory functions (29, 30), with low levels indicating malnutrition and systemic inflammation that are linked to poor pneumonia outcomes (31–34). Lymphopenia, conversely, indicates impaired cellular immunity (36) and has been consistently associated with higher mortality in severe infections (13, 35, 39, 40). By integrating these markers, PNI provides a comprehensive measure of host vulnerability, where a lower score reflects both nutritional depletion and immune dysfunction, thereby explaining its strong association with short-term mortality (6, 26, 41). The combination of these two parameters, known as the Prognostic Nutritional Index (PNI), which overcomes this limitation by offering a comprehensive reflection of both the nutritional status and immune function of the body, enhancing the ability to predict the prognosis in pneumonia patients effectively (6), and it was considered more reliable than either albumin levels or lymphocyte counts alone. Malnutrition is linked to adverse clinical outcomes in numerous diseases, such as cardiovascular disease (42, 43), liver disease (44), pulmonary disease (45, 46)and so on. According to a systematic review of 29 observational study that poor nutritional status (OR, 6.14; 95% CI, 0.65–11.58) is one of the risk factors for pneumonia (47). In a retrospective study with 450 patients aged between 38 and 78, PNI was superior to neutrophil-to-lymphocyte ratio (NLR) in predicting mortality (41). A study of Wang et al. identified that PNI had a significant negative association with the risk of mortality in patients with CAP (6), which is consistent with our findings. However, their study population was from the United States, which is different from the Chinese population in our study. The study of association between PNI with the Mortality in pneumonia patients on Asian populations, especially on the Chinese population, are relatively rare. Our study focuses on hospitalized pneumonia patients in China, and these patients with low PNI scores upon hospital admission are likely to experience poorer outcomes. Therefore, the stratification of nutritional risk, combined with timely nutritional intervention strategies, is crucial for improving hospitalization outcomes. The research had showed that early high nutritional support can improve outcomes for critically ill patients (48), which emphasizing the importance of prioritizing nutritional interventions early. During hospitalization, patients were provided with a high-protein diet or supplements to improve immune function and overall nutritional status. Moreover, repeated assessments of the Prognostic Nutritional Index (PNI) were conducted to guide the monitoring of nutritional biomarkers and the adjustment of nutritional support strategies. Implementing interventions targeting specific risks associated with low PNI can help hospitalized patients reduce the risk of adverse outcomes, thereby improving the prognosis of patients with pneumonia.

In summary, PNI represents a simple, cost-effective, and widely available index that may help clinicians identify pneumonia patients at higher risk of poor outcomes. Its integration into routine clinical practice could support early risk stratification and personalized management.

Naturally, this study has several potential limitations. First, as a retrospective cohort study, it is inherently limited in establishing a definitive causal relationship between PNI levels and the progression of pneumonia. Second, the retrospective design may introduce biases and confounding factors inherent to observational research, including selection bias. Despite multivariable adjustments, unmeasured or residual confounders may still exist, such as nutritional interventions, socioeconomic status, and comorbidities not included in the analysis, which could affect the reliability of the findings. Third, since PNI can be influenced by pathological conditions unrelated to pneumonia, its application as a prognostic indicator should be interpreted with caution. Fourth, the original data were derived from multiple centers, and no stratified analysis was conducted based on hospital sites; moreover, the choice of tertile cutoffs may also limit the generalizability of the findings to broader healthcare settings.

## 5 Conclusion

In conclusion, in this multicenter retrospective cohort study, we demonstrated that lower PNI levels were significantly associated with higher mortality in patients with pneumonia. The strengths of this study include the use of a large multicenter dataset with sufficient statistical power, the investigation of PNI in pneumonia outcomes within a Chinese cohort, and the application of robust statistical modeling methods such as Cox regression, restricted cubic splines, and subgroup analyses. These findings suggests that PNI may serve as a simple, cost-effective and practical biomarker for risk stratification in clinical practice. By integrating both nutritional and immunological status, PNI provides clinicians with an accessible indicator to identify high-risk patients and optimize management strategies. Further multicenter, large-sample prospective studies are needed to validate our findings and to explore the underlying biological mechanisms.

## Data Availability

Publicly available datasets were analyzed in this study. This data can be found here: Repository Name: Dryad Digital Repository Direct URL: https://doi.org/10.5061/dryad.mkkwh70x2.

## Footnotes

^1^ https://datadryad.org/dataset/doi:10.5061/dryad.mkkwh70x2

## Glossary

PNI: The Prognostic Nutritional Index
HBP: Hypertension
DM: Diabetes mellitus
SBP: Systolic pressure
DBP: Diastolic pressure
MV: Mechanical Ventilation
PSI: Pneumonia Severity Index
LAC: Lactate
WBC: White blood cell
NUET: Neutrophils
LYM: Lymphocyte
HGB: Hemoglobin
PLT: Platelet
ALB: Albumin
AST: Aspartate aminotransferase
ALT: Alanine aminotransferase
BUN: Blood Urea Nitrogen
Cr: Creatinine
K^+^: Potassium
Na^+^: Sodium
NLR: neutrophil-to-lymphocyte ratio
ref.: reference
