# Supplementary material for: Prognostic nutritional index and mortality in pneumonia: a retrospective cohort study in China
Source: Front Nutr. 2025 Oct 15;12:1660457. doi: 10.3389/fnut.2025.1660457 (PMC12568431; doi:10.3389/fnut.2025.1660457)
Supplement: Supplementary file 1 [file Data_Sheet_1.zip › Table S1 Univariate regression analysis.docx]

**Supplementary Table1** Univariate regression analysis (30-day and 90-day all-cause mortality)

| Variables | 30-day mortality | | 90-day mortality | |
| --- | --- | --- | --- | --- |
|  | HR (95%CI) | P value | HR (95%CI) | P value |
| Age (cont. var.) | 1.01 (1,1.02) | 0.036 | 1.01 (1,1.02) | 0.006 |
| Gender: Female vs male | 0.96 (0.69,1.33) | 0.811 | 0.93 (0.69,1.26) | 0.64 |
| HBP: Yes vs No | 1.08 (0.77,1.51) | 0.675 | 1.11 (0.81,1.52) | 0.525 |
| DM: Yes vs No | 1.11 (0.77,1.59) | 0.588 | 1.18 (0.84,1.65) | 0.333 |
| Respiratory Failure (cont. var.) | 34.92 (14.3,85.25) | < 0.001 | 35.36 (15.64,79.92) | < 0.001 |
| Temperature (cont. var.) | 1.35 (1.17,1.56) | < 0.001 | 1.35 (1.18,1.55) | < 0.001 |
| Heart rate (cont. var.) | 1.0084 (1.0009,1.016) | 0.029 | 1.0054 (0.9985,1.0122) | 0.125 |
| Respiratory rate (cont. var.) | 1.03 (1.02,1.05) | < 0.001 | 1.03 (1.02,1.05) | < 0.001 |
| SBP (cont. var.) | 1.002 (0.9935,1.0106) | 0.647 | 1.0018 (0.9939,1.0099) | 0.654 |
| DBP (cont. var.) | 0.9931 (0.9802,1.0063) | 0.304 | 0.9914 (0.9793,1.0036) | 0.166 |
| MV: Yes vs No | 11.08 (7.24,16.96) | < 0.001 | 13.56 (8.96,20.51) | < 0.001 |
| Invasive ventilation: Yes vs No | 6.83 (4.87,9.58) | < 0.001 | 7.92 (5.76,10.89) | < 0.001 |
| Noninvasive ventilation: Yes vs No | 5.13 (3.69,7.13) | < 0.001 | 5.72 (4.2,7.79) | < 0.001 |
| PSI (cont. var.) | 1.02 (1.01,1.02) | < 0.001 | 1.02 (1.01,1.02) | < 0.001 |
| LAC (cont. var.) | 1.21 (1.13,1.29) | < 0.001 | 1.21 (1.13,1.28) | < 0.001 |
| WBC (cont. var.) | 1.05 (1.03,1.07) | < 0.001 | 1.05 (1.03,1.07) | < 0.001 |
| NUET (cont. var.) | 1.02 (1.01,1.03) | < 0.001 | 1.02 (1.01,1.03) | < 0.001 |
| LYM (cont. var.) | 0.64 (0.49,0.84) | 0.001 | 0.65 (0.51,0.84) | < 0.001 |
| HGB (cont. var.) | 0.992 (0.9848,0.9992) | 0.03 | 0.9912 (0.9845,0.9979) | 0.01 |
| PLT (cont. var.) | 0.9957 (0.9937,0.9978) | < 0.001 | 0.9959 (0.994,0.9977) | < 0.001 |
| ALB (cont. var.) | 0.92 (0.89,0.94) | < 0.001 | 0.92 (0.9,0.94) | < 0.001 |
| AST (cont. var.) | 1.0018 (1.0012,1.0024) | < 0.001 | 1.0018 (1.0012,1.0024) | < 0.001 |
| ALT (cont. var.) | 1.0033 (1.0017,1.0049) | < 0.001 | 1.0031 (1.0015,1.0047) | < 0.001 |
| BUN (cont. var.) | 1.04 (1.03,1.06) | < 0.001 | 1.05 (1.03,1.06) | < 0.001 |
| Cr (cont. var.) | 1.0012 (0.9999,1.0025) | 0.078 | 1.001 (0.9997,1.0023) | 0.148 |
| K^+^ (cont. var.) | 1.04 (1.02,1.05) | < 0.001 | 1.04 (1.02,1.05) | < 0.001 |
| Na^+^ (cont. var.) | 0.96 (0.93,0.99) | 0.004 | 0.95 (0.92,0.98) | 0.001 |
| Vasoactive drugs: Yes vs No | 8.44 (6.06,11.75) | < 0.001 | 9.8 (7.18,13.39) | < 0.001 |
| High-dose glucocorticoid: Yes vs No | 2.04 (1.47,2.84) | < 0.001 | 2.07 (1.53,2.81) | < 0.001 |
| Immunosuppressant: Yes vs No | 0.98 (0.7,1.37) | 0.911 | 0.98 (0.72,1.34) | 0.9 |
| Ganciclovir: Yes vs No | 2.19 (1.55,3.08) | < 0.001 | 2.21 (1.61,3.04) | < 0.001 |
| Sulfonamide: Yes vs No | 1.93 (1.38,2.69) | < 0.001 | 2.03 (1.48,2.78) | < 0.001 |
| Anti-Aspergillus: Yes vs No | 2.49 (1.78,3.48) | < 0.001 | 2.9 (2.12,3.98) | < 0.001 |
| Anti-Pseudomonas: Yes vs No | 8.09 (3.57,18.31) | < 0.001 | 8.2 (3.85,17.48) | < 0.001 |
| PNI (cont. var.) | 0.93 (0.9,0.95) | < 0.001 | 0.93 (0.91,0.95) | < 0.001 |
| PNI3: ref.=Tertile 1 |  |  |  |  |
| Tertile 2 | 0.39 (0.26,0.57) | < 0.001 | 0.42 (0.29,0.6) | < 0.001 |
| Tertile 3 | 0.21 (0.13,0.34) | < 0.001 | 0.24 (0.16,0.37) | < 0.001 |

Notes: HBP, Hypertension; DM, Diabetes mellitus; SBP, Systolic pressure; DBP, Diastolic pressure; MV, Mechanical Ventilation; PSI, Pneumonia Severity Index; LAC, Lactate; WBC, White blood cell; NUET, Neutrophils; LYM, Lymphocyte; HGB, Hemoglobin; PLT, Platelet; ALB, Albumin; AST, Aspartate aminotransferase; ALT, Alanine aminotransferase; BUN, blood urea nitrogen; Cr, Creatinine; K^+^, Potassium; NA^+^, Sodium; PNI, Prognostic Nutritional Index; ref., reference
