# Supplementary figures and images for: Prognostic nutritional index and mortality in pneumonia: a retrospective cohort study in China
Source: Front Nutr. 2025 Oct 15;12:1660457. doi: 10.3389/fnut.2025.1660457 (PMC12568431; doi:10.3389/fnut.2025.1660457)

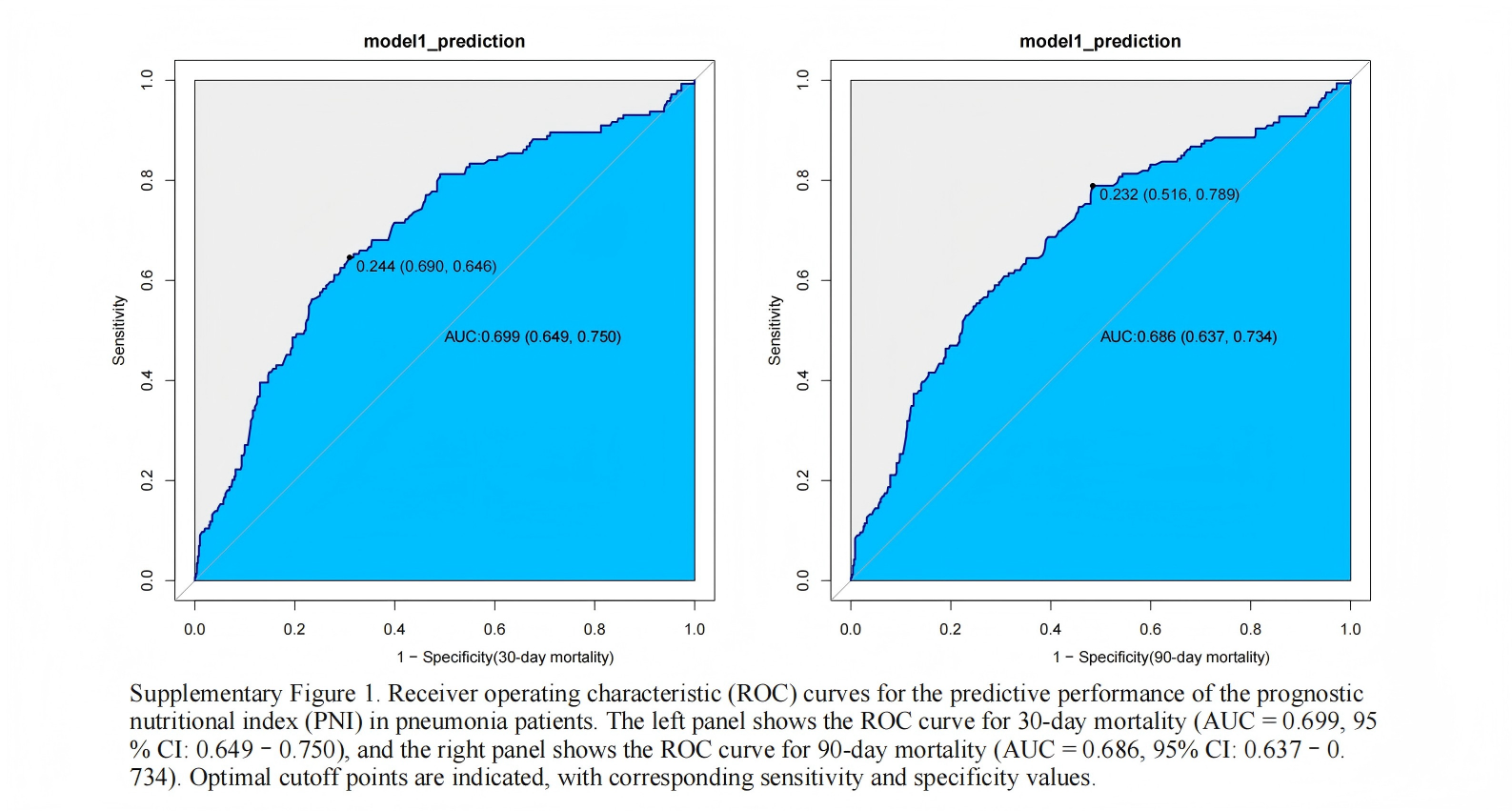

Supplement: Supplementary file 1 [file Data_Sheet_1.zip › figure S1.ROC analysis.tiff]
